# Faecal carriage of CTX-M extended-spectrum beta-lactamase-producing Enterobacteriaceae among street children dwelling in Mwanza city, Tanzania

**DOI:** 10.1371/journal.pone.0184592

**Published:** 2017-09-12

**Authors:** Nyambura Moremi, Heike Claus, Ulrich Vogel, Stephen E. Mshana

**Affiliations:** 1 Department of Microbiology and Immunology, Catholic University of Health and Allied SciencesBugando, Mwanza, Tanzania; 2 Institute for Hygiene and Microbiology, University of Wuerzburg, Wuerzburg, Germany; Robert Koch Institute, GERMANY

## Abstract

**Background:**

Data on ESBL carriage of healthy people including children are scarce especially in developing countries. We analyzed the prevalence and genotypes of ESBL-producing Enterobacteriaceae (EPE) in Tanzanian street children with rare contact to healthcare facilities but significant interactions with the environment, animals and other people.

**Methodology/ Principle findings:**

Between April and July 2015, stool samples of 107 street children, who live in urban Mwanza were analyzed for EPE. Intestinal carriage of EPE was found in 34 (31.8%, 95% CI; 22.7–40.3) children. Of the 36 isolates from 34 children, 30 (83.3%) were *Escherichia coli* (*E*. *coli*) and six *Klebsiella pneumoniae* (*K*. *pneumoniae*). Out of 36 isolates, 36 (100%), 35 (97%), 25 (69%) and 16 (44%) were resistant to tetracycline, trimethoprim-sulfamethoxazole, ciprofloxacin and gentamicin, respectively. Beta-lactamase genes and the multilocus sequence types of *E*. *coli* and *K*. *pneumoniae* were characterized. ESBL gene *bla*_CTX-M-15_ was detected in 75% (27/36) of ESBL isolates. Sequence types (STs) 131, 10, 448 and 617 were the most prevalent in *E*. *coli*. Use of local herbs (OR: 3.5, 95% CI: 1.51–8.08, *P* = 0.003) and spending day and night on streets (OR: 3.6, 95% CI: 1.44–8.97, *P* = 0.005) were independent predictors of ESBL carriage.

**Conclusions/ Significance:**

We observed a high prevalence of *bla*_CTX-M-15_ in EPE collected from street children in Tanzania. Detection of *E*. *coli* STs 131, 10, 38 and 648, which have been observed worldwide in animals and people, highlights the need for multidisciplinary approaches to understand the epidemiology and drivers of antimicrobial resistance in low-income countries.

## Introduction

Extended-spectrum Beta-lactamases (ESBLs) are enzymes produced by Gram-negative bacteria that mediate resistance to penicillins, cephalosporins and monobactams [1]. ESBL-producing Enterobacteriaceae (EPE), particularly *Escherichia coli* (*E*. *coli*) and *Klebsiella pneumoniae* (*K*. *pneumoniae*), have been linked to healthcare-associated infections, but are repeatedly isolated from community-acquired bacterial infections [2–4]. Previous studies reported that the colonization by ESBL-producing organisms was a potential risk factor for EPE infections [5, 6]. ESBL-producing organisms are associated with multidrug-resistant infections in children [7]. In Mwanza, Tanzania, ESBL-producing bacteria are increasingly reported as clinical isolates and are associated with increasing morbidity and mortality [8, 9]. A high prevalence of EPE carriage has been reported in hospitals and communities worldwide, particularly in low- and middle-income countries [10, 11]. A recent study of healthy people in Mwanza community reported the faecal EPE carriage rate of 16.5% [12]. High faecal ESBL carriage ranging from 10% to 39% have been reported among healthy and hospitalized children in Africa [13, 14].

To our knowledge street children have never been evaluated for carriage of multidrug resistant organisms (MDRO), despite the fact that; (i) street children play along Mirongo river and majority swim in Lake Victoria which are areas of the city known to be contaminated with EPE, and have extensive interaction with other people and animals [15]; (ii) knowledge about the prevalence of MDRO is important for empirical management of infections in this neglected cohort; (iii) due to their limited contact with medical care, finding MDRO in street children indicates that community-level spread of MDRO is occurring. We therefore analyzed the prevalence of EPE in more than 100 street children living in Mwanza city to provide data on the epidemiology of the EPE in this special group prevalent in developing countries.

## Materials and methods

### Ethics approval

The study protocol was approved by the Joint CUHAS/Bugando Medical Centre ethics and scientific review committee (CREC 073/2015). The Mwanza city social welfare department granted the permission of conducting a study in street children. Study goals were explained to children and those who agreed to participate were asked questions regarding their demographics, street lifestyles and other factors that might be associated with ESBL carriage.

### Study design, site and enrollment of participants

A cross-sectional study was performed between April and July 2015. A total of 300 street children are estimated to reside in Mwanza city. As per 2012 national census, the Mwanza region has a population of 2,772,509 within an area of 9,467 km^2^. Out of 226 children who attended a newly found healthcare camp, 107 children were willing to participate and were serially enrolled to the study. Using a structured survey instrument, children were interviewed for their street lifestyle, which was categorized to type 1 “on street children” (those who spent the day on streets and at night they sleep either at home or in street children institutions) and type 2 “of street children” (those who spent the day and sleep on streets). Other information such as age, sex, history of being into contact with modern healthcare, use of local herbs and their source of food were also recorded. Because most of these children had no formal education, which could potentially contribute to recall bias, antibiotic use history within the past 4 weeks was assessed by showing them amoxicillin capsules commonly known as “rangi-mbili” in the community. A general examination was done including taking anthropometric measurements. In addition, blood glucose levels were measured by using finger-prick test (Glucoplus Inc, Canada).

### Sample collection

Every participant was instructed on how to collect a stool sample and was provided with a clean scooped-plastic container (Paul Böttger oHG, Germany) for this purpose. A total of 107 non-repetitive collected stool specimens were kept in a cool box and transported to CUHAS laboratory for microbiological analysis within 4 hours of collection.

### Laboratory procedures and ESBL detection

Using sterile disposable loops (Sarstedt AG & Co, Germany), stool specimens were inoculated on MacConkey agar (BD Difco, USA) supplemented with 2μg/ml of cefotaxime (Medochemie Ltd, Cyprus EU). Plates were incubated at 37°C for 24 hours. Predominant colonies of different morphotypes were identified to species level by using in-house biochemical tests (Triple Sugar Iron Agar, Sulfur-Indole-Motility test, Simmons' citrate Agar, and Urease test) as previously described [16]. Screening for ESBL and identification of EPE was done using CHROMagar™ ESBL (Mast Diagnostica GmbH, Germany) by observing pink-reddish (*E*. *coli*) and metallic-blue (*K*. *pneumoniae*) colonies. *E*. *coli* ATCC 25922 (non-beta-lactamase-producing strain) and *E*. *coli* ATCC 35218 (beta-lactamase-producing strain) were used for quality control. Isolates were frozen in ready-to-use Microbank^TM^ beads (Pro-Lab Diagnostics, U.K) for further molecular analysis at the Institute for Hygiene and Microbiology (IHM), Wuerzburg, Germany.

At IHM the EPE isolates were re-cultured. Antimicrobial susceptibility and ESBL production testing were done by using an automated VITEK®-2 system (bioMérieux, France). ESBL confirmation was done by disc approximation method. European Committee on Antimicrobial Susceptibility Testing minimum inhibitory concentration breakpoints were used to classify isolates to either susceptible or resistant [17].

### ESBL gene analysis

Primers CTX-M3G-F (5’-GTTACAATGTGTGAGAAGCAG-3’) and CTX-M3G-R (5’-CCGTTTCCGCTATTACAAAC-3’) were used in PCR to amplify the complete *bla*_CTX-M_ gene for CTX-M group I as previously described [18]. For the isolates that were negative for CTX-M group I, additional primers for CTX-M groups II, III, IV, V, SHV and TEM genes were performed to identify other ESBL alleles as described by Shi *et al*., [19]. PCR conditions for all primer pairs were: initial denaturation at 94°C for 10 min, denaturation at 94°C for 1 min, annealing at 55°C for 1 min, and elongation at 72°C for 1 min, repeated for 35 cycles; and a final extension at 72°C for 10 min. The PCR reaction mixture was carried out in a 50-μl volume consisting of 200nMof each primer, 200 μM deoxynucleoside triphosphates, 2mM MgCl_2_ provided with the ThermoPol® Buffer, and 1 U of the Taq polymerase (New England Biolabs Ltd, UK). In each PCR run a negative control was used to test for possible laboratory contamination and a positive control was used to control for false negatives. Purification and double-stranded sequencing of the PCR products was done at LGC Genomics GmbH (Berlin, Germany). *Bla*_CTX-M_, SHV and TEM gene sequences of respective isolates were assembled, trimmed and quality control checked using SeqMan Pro software (DNASTAR Inc. USA). The NCBI blastn algorithm [20] was used to identify the ESBL alleles of the consensus sequences.

### Multilocus sequence typing

All ESBL-producing isolates were subjected to multilocus sequence typing (MLST). The PCR template was prepared by boiling a suspension of 1 to 2 bacterial colonies in 1 ml of distilled water for 15 min, followed by centrifugation at 13,000 rpm for 1 min. The MLST scheme developed by Wirth *et al*., [21] was used for *E*. *coli* while the MLST scheme of Diancourt *et al*.,[22] was used for *K*. *pneumoniae*. The PCR products were sequenced at LGC Genomics GmbH (Berlin, Germany). TraceEditPro (Ridom GmbH, Germany) was used to analyze the sequences and isolates were assigned to their respective sequence types according to http://mlst.warwick.ac.uk/mlst/dbs/Ecoli and http://bigsdb.pasteur.fr/klebsiella/klebsiella.html, MLST databases for *E*. *coli* and *K*. *pneumoniae*, respectively.

### Phylogenetic analysis

MEGA-4 software [23] was used to generate a neighbour joining tree to infer evolutionary relationships between the *E*. *coli* STs. The analysis was done by using concatenated MLST gene sequences of the *E*. *coli* STs. Bootstrap percentages were calculated from 500 replicates.

### Data management and statistical analysis

Laboratory journals were used to record participants’ demographic data, laboratory experiments and results. Data were then transferred to a Microsoft Excel spreadsheet (Microsoft Corporation, USA). Categorical variables were summarized as percentages or proportions. Median with interquartile ranges and means with standard deviations were used to summarize continuous variables depending on the distribution of data. Pearson’s Chi-square and Fischer’s exact tests were used to observe the statistical differences among various categories. Univariate regression analysis was employed followed by multivariate analysis for the factors with *P*<0.05 to identify factors that were associated with ESBL carriage. Data were analyzed using STATA-13 software (STATA Corp LP, USA) and *P* ≤ 0.05 was used for a significance threshold.

## Results

### Demographics and clinical parameters

A total of 107 street children (98.1% male) with the age range between 7 and 17 years were enrolled. The mean age of the participants was 14.2 ± 3.6 years. Type 2 children, i.e. those who spent the day and slept on streets, contributed to 58.9% (63/107) of the total number. The mean Body Mass Index (BMI) of these children was 18.27 ± 2.26 kg/m^2^ while the mean blood glucose level was 4.82 ± 0.77 mmol/L. Of 107 children, 10 (9.3%) completed primary education, 60 (56.1%) dropped off while 37 (34.6%) did not attend the primary education at all. Eighty-seven children (81.3%) had living parents. Of 107 children, 30 (28%) used antibiotics in the past 4 weeks before enrolment.

### ESBL carriage rate and phenotypic resistance patterns

Of 107 children, 34 (31.8%, 95% CI; 22.7–40.3) were colonized with at least one ESBL-producing *E*. *coli* or *K*. *pneumoniae* isolate. Double colonization of *E*. *coli* and *K*. *pneumoniae* isolates was detected in two faecal samples making a total number of isolates 36. Of the 36 ESBL-producing isolates detected, 83.3% (30/36) and 16.7% (6/36) were *E*. *coli* and *K*. *pneumoniae*, respectively. All 36 ESBL isolates were resistant to tetracycline, while resistance rates to trimethoprim-sulfamethoxazole, ciprofloxacin and gentamicin were 97% (35/36), 69% (25/36) and 44% (16/36), respectively, Table 1. Co-resistance of ESBL isolates to trimethoprim-sulfamethoxazole, ciprofloxacin and gentamicin was detected in 12 (33.3%) of the 36 isolates. Tigecycline resistance was detected in four out of six *K*. *pneumoniae* isolates. All isolates were susceptible to colistin, and meropenem.

**Table 1:** Proportion of antimicrobial resistance and beta-lactamase genes among 36 ESBL producing *E*. *coli* and *K*. *pneumoniae*.

Antimicrobial agent	*E*. *coli* (N = 30) %	*K*. *pneumoniae* (N = 6) %
Ciprofloxacin	70.0	66.7
Gentamicin	33.3	100
Moxifloxacin	80.0	100
Piperacillin-Tazobactam	100	100
Tetracycline	100	100
Tigecycline	0.0	66.7
Trimethoprim-Sulfamethaxozale	96.7	100
MDRO^a^ (SXT, CIP, GEN)^b^	26.7	66.7
MDRO^a^ (SXT, CIP, GEN, TGC)	0.0	50.0
***Bla* genes**	**n**	**n**
CTX-M-15	23	4
CTX-M-9	5	2
CTX-M-55	2	-
TEM-1	19	6
SHV-1	-	3
SHV-11	-	3

^**a**^MDRO: resistant to at least one agent in three or more antimicrobial categories [24].

^**b**^SXT: trimethoprim-sulfamethoxazole, CIP: ciprofloxacin, GEN: gentamicin, TGC: tigecycline

### ESBL enzymes

All 36 ESBL isolates carried CTX-M genes. Twenty-seven (75%) harboured the *bla*_CTX-M-15_ allele [*E*. *coli* (23) and *K*. *pneumoniae* (4)] and two *E*. *coli* isolates (5.6%) the *bla*_CTX-M-55_ allele. The remaining seven (19.4%) isolates [*E*. *coli* (5) and *K*. *pneumoniae* (2)] were PCR-positive for CTX-M-9 group. Non-ESBL TEM-1 was detected in 25 (69.4%) [*E*. *coli* (19) and *K*. *pneumoniae* (6)] of the 36 ESBL isolates, whereas non-ESBL SHV were detected in all six *K*. *pneumoniae* isolates [SHV-1 (3), SHV-11(3)].

### Sequence types and phylogenetic analysis

**Fig 1:**
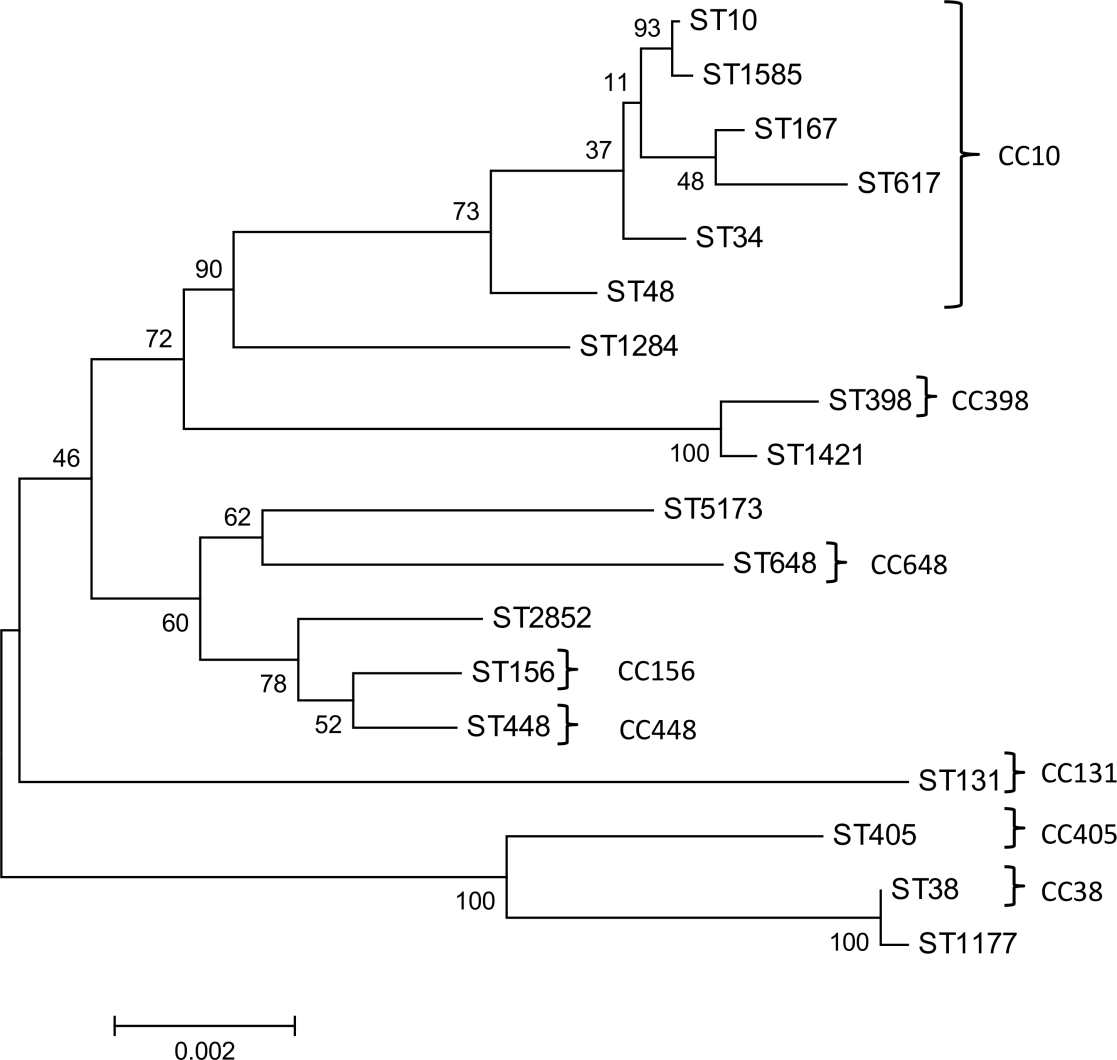
Neighbor joining tree of concatenated gene sequences of the sequence types (ST) identified in ESBL-producing *E*. *coli*. The 30 *E*. *coli* isolates were assigned to 18 STs. Clonal complexes (CC) were shown where applicable. The tree was generated with MEGA 4. The bootstrap values based on 500 replicates. The scale bar (0.002) indicates the number of nucleotide substitutions per site.

Eighteen sequence types (STs) were observed in 30 ESBL-producing *E*. *coli* of which ST131 (5/30), ST10 (3/30), ST448 (3/30) and ST617 (3/30) were the most prevalent. The STs could be assigned to eight clonal complexes with six STs belonging to CC10. The phylogenetic analysis revealed *E*. *coli* strains to be very diverse (Fig 1). The six *K*. *pneumoniae* isolates were assigned to ST1 (3/6), ST261 (2/6) and ST54 (1/6).

### Predictors of ESBL carriage

Higher ESBL carriage was observed among children who used local herbs for paramedical purposes compared with those who did not use herbs (50% vs. 22.2%, *P* = 0.003), Table 2. When categorized by herbal type, those who either drank Aloe vera and unknown root juice or who chewed roots had a higher prevalence of ESBL compared to those who took Neem leaves (*Azadirachta indica*) (60.9% v. 33.3%, *P* = 0.004). Univariate analysis indicated that ESBL carriage was significantly higher in type 2 street children (OR: 3.6, 95% CI 1.4–8.7, *P* = 0.005), Table 2. By multivariate logistic regression analysis only local herbal use (OR: 3.3, 95% CI 1.31–8.31, *P* = 0.011) and street child type (OR: 2.8, 95% CI 1.04–7.65, *P* = 0.041) were independent predictors of ESBL carriage. There was no significant association of ESBL carriage in children who reported having used antibiotics and those who did not report use (33.3% vs. 33.7%, *P* = 0.966). There was no association between either source of food or education level and EPE carriage, Table 2.

**Table 2:** Predictors of ESBL carriage among street children in Mwanza city.

Variable	All children	ESBL Positive N = 36	Univariate OR (95% CI)	*P* value	Multivariate OR (95% CI)	*P* value
**Age (years)**	14.2 ± 3.62	14.5 ± 3.29	1.03 (0.92–1.15)	0.549		
**Primary education**						
Not attended	37	11 (29.7%)	1			
Incomplete	60	22 (36.7%)	1.36 (0.12–3.67)	0.483		
Completed	10	3 (30%)	1.01 (0.59–2.16)	0.986		
**Local herbal use**						
No	63	14 (22.2%)	1			
Yes	44	22 (50%)	3.49 (1.51–8.08)	0.003	3.1 (1.31–7.41)	0.011
**Source of income**						
Selling scrap metals	24	6 (25%)	1			
Selling plastic bottles	64	30 (46.9%)	2.64 (0.92–7.53)	< 0.001	0.7 (0.34–1.56)	0.424
Begging	19	0 (0%)	-			
**Source of food**						
Buy	81	25 (30.9%)	1			
Scavenging	26	11 (42.3%)	1.64 (0.66–4.07)	0.283		
**Street child type**						
Type 1	44	8 (18.2%)	1			
Type 2	63	28 (44.4%)	3.6 (1.44–8.97)	0.005	3 (1.17–7.81)	0.022

## Discussion

Data from the present study show a high prevalence of faecal carriage of EPE among street children residing in Mwanza city. Despite the fact that there is no comparative group, the observed prevalence of 31.8% in this study is significantly higher (*P*≤0.001) compared to 16.5% observed in a recent study in the same city among healthy people [12]. The observed prevalence in street children is also higher than 11.6% recently observed among healthy children in the Dar-es-salaam region in Tanzania [25]. The mechanism responsible for higher prevalence is not clear. On the one hand, these children spend most of their time playing in areas of the city reportedly contaminated with EPE and heavy metals [15]. Furthermore, the consumption of food leftovers that might have been contaminated with birds/animal faeces carrying EPE could potentially contribute to a higher prevalence of EPE carriage in this neglected group [26]. This argument is further supported by the fact that in the current study children who scavenged food leftovers were more likely to be colonized with EPE.

We found no association between antibiotic use and EPE carriage. This surprising finding might be explained by the poor quality of the self-reported data due to the type of assessment and poor recall of information for long duration as recently reported in Kenya, a neighboring East African country [27]. Another probable reason might be due to social acceptability bias (children think that they are not supposed to take antibiotics on their own, therefore cannot report). Nevertheless, the high number of children reporting antibiotic use despite rare contact to the healthcare system is worrying and requires further investigation. On the contrary, the use of local herbs was predictive of EPE carriage. Poor hygienic conditions either in preparing these herbs or the environment where these plants grow might have led to the contamination with EPE as previously observed in culinary herbs that were predominantly contaminated with EPE harbouring variants of CTX-M enzymes [28]. This is related to transmission of ESBL genes to people via the food chain [29]. In addition, these herbs might have antibacterial effect; however, this was not investigated in this study.

Type 2 street children were more frequently colonized with EPE compared to type 1 street children. The lifestyle of type 2 children makes them more vulnerable to health problems because they spend their entire day on streets, scavenge food leftovers, contact possibly contaminated environment, and sleep in overcrowded unhygienic places as previously observed [30, 31].

ST131 was a predominant sequence type among the *E*. *coli* isolates. ST131 isolates harbouring *bla*_CTX-M-15_ are distributed across the globe and have been isolated [32–34]. The ability of these birds to move from one place to another while feeding on garbage and leaving their droppings in the environment, which these children are more likely to live in, might explain the carriage of this pandemic clone in street children with rare visit to the healthcare. ST10 and ST617, that belong to clonal complex 10 previously reported as predominant clones in Nigeria [35] were also prevalent in this study, and have been detected in animals [36] and healthy people [12] in the same city where the current study took place. These findings indicate the similarity of strains across different interfaces within the same region and Africa in general. Furthermore, ST448, an internationally disseminated clone previously reported in non-ESBL *E*. *coli* in countries like Nigeria, Thailand, India and Germany and recently in ESBL-producing *E*. *coli* in Norway [37] was also detected in our study. Cephalosporin-resistant CTX-M-15-producing *K*. *pneumoniae* of ST1, which has been reported to emerge and spread in different places including Europe [38], was also common in street children in the city of Mwanza, Tanzania; connoting the epidemiological spread of this clone.

We isolated *E*. *coli* ST1177 carrying *bla*_CTX-M-55_ from a street child. In a previous study, the related ST1177 *E*. *coli* isolate SO042, which had similar phenotypic characteristics, was found in the muddy soil around Mirongo river; a common place in the city of Mwanza where the majority of these children play [39]. Previous study in the same setting showed high proportion of samples along the river were positive for EPE [39]. There is high possibility that the river is contaminated by sewage because the municipal sewage treatment does not service the entire community in the city especially those residing in squatters making these children more likely to be exposed to faecal waste. Furthermore, these children bath and swim in the Lake Victoria which receives biologically treated wastewater effluent from Mwanza city and all other wastes especially during rainy season. These findings point to the possible role played by the environment in dissemination of MDRO as it has been observed to contain a complex mixture of faecal materials from people and animals [40].

## Conclusion

A high faecal carriage of EPE harbouring *bla*_CTX-M-15_ was observed among street children residing in Mwanza city. This information is of public health importance because it points to the existence of multidrug-resistant Enterobacteriaceae even in cohorts with presumably very limited access to medical care or antibiotics. The presence of overlapping clones of *E*. *coli* in animals, environment and people in the city of Mwanza calls for a multidisciplinary approach to combat the upsurge of antimicrobial resistance in low-income countries.
